# Longitudinal Analysis of Serum Autoantibody-Reactivities in Patients with Primary Open Angle Glaucoma and Optic Disc Hemorrhage

**DOI:** 10.1371/journal.pone.0166813

**Published:** 2016-12-28

**Authors:** Katrin Lorenz, Sabine Beck, Munir M. Keilani, Joanna Wasielica-Poslednik, Norbert Pfeiffer, Franz H. Grus

**Affiliations:** 1 Department of Experimental Ophthalmology, University Medical Center, Johannes Gutenberg-University, Mainz, Germany; 2 Department of Pharmacy and Biochemistry, Johannes Gutenberg-University Mainz, Germany; 3 Vision 100 Die Augenärzte, Gemeinschaftspraxis Mönchengladbach, Germany; University of Iowa, UNITED STATES

## Abstract

**Background:**

The aim of our current investigation was to analyze the autoantibody-reactivities of primary open angle glaucoma patients with optic disc hemorrhage as possibly correlated to disease progression by means of a protein microarray approach.

**Methods:**

Sera of patients with primary open angle glaucoma and optic disc hemorrhage (n = 16) were collected directly after study inclusion (0 weeks) and after 2 weeks, 4 weeks and 12 weeks. As a control group patients with primary open angle glaucoma (n = 18) were used (0 weeks and 12 weeks). Microarrays were incubated and occurring antibody-antigen-reactions were visualized with fluorescence labeled anti-human-IgG secondary antibodies. To detect changes in autoantibodies spot intensities were digitized and compared.

**Results:**

With respect to the immunoreactivity at 0 weeks level increment of anti-adaptor protein 1 complex subunit mu-1 antibodies and anti-SPRY domain-containing SOCS box protein 3 antibodies in sera of primary open angle patients with optic disc hemorrhage was detected. Linear trend analysis revealed a positive correlation with r ≥ 0.8 between antibody-level and time course. Control group show no relevant changes in the same period. Significant changes were found in time point 4 comparison between patient groups in anti-adaptor protein 1 complex subunit mu-1-level (p = 0.01). No significant changes in visual acuity were found.

**Conclusion:**

With this approach we were able to detect autoimmune reactivities in sera of patients with primary open angle glaucoma and optic disc hemorrhage compared to patients without optic disc hemorrhage. These antibodies could give further insights into the pathogenesis and the autoimmune component of glaucomatous optic neuropathy.

## 1. Introduction

Glaucoma is the first cause for inalterable visual impairment and irreversible blindness worldwide [1]. In 2040 the number of persons concerned is estimated about 111 million. Glaucoma is a disease of multi-factorial origin with glaucomatous optic neuropathy (GON) and progressive degeneration of retinal ganglion cells (RGC) as major characteristics [2, 3]. Among other glaucoma types primary open angle glaucoma (POAG) is the most common form with a global prevalence of 3% [4, 5]. Elevated intraocular pressure (IOP) is known as the major risk factor and topical pressure lowering therapy is still the first-line treatment in glaucoma disease management. But until now the detailed mechanisms of occurring RGC loss remain unclear [6]. Beside dysfunctional vascular regulation, reactive nitrogen and oxygen species, impaired mitochondria and other factors an autoimmune component is discussed as possible part of glaucoma pathogenesis [7–11]. Also antibodies (abs) have emerged in the research focus as potential players in GON. In several studies autoabs and complex autoab-profiles could be detected in body fluids of patients with glaucoma compared to healthy subjects [12–15]. Furthermore, not only up-regulated, but also down-regulated abs are shown in glaucoma affected individuals [16]. Silent onset, slow and unnoticed progression of visual field narrowing makes diagnosis of the disease challenging in daily clinical routine. Therefore occurring RGC loss and subsequent defects in the optic nerve head are mostly detected when almost 20–40% of RGC are irreversible lost [17–19]. Irrecoverable visual decay is often the consequence and applied treatment can only slow down or halt pathologic progression. Optimized detection of progression would be strongly beneficial and could ameliorate medical maintenance of patients [20]. This underlines the strong demand for better diagnosis of early glaucoma stages and subsequent structural damage. A widely accepted indication for development and progression of glaucoma is optic disc hemorrhage (ODH). This splinter-shaped area of bleeding has found to be associated with the likelihood of disease progression in different large studies and has been considered as an important feature of changes in GON [21–26]. To support clinically suspect glaucoma cases an easy to use protein microarray approach with predictive autoabs displaying an upcoming structural worsening could be helpful. Therefore this study aims to analyze autoab-profiles in POAG patients with ODH as a sign of ongoing glaucoma progression in comparison to POAG subjects without ODH. The detection of autoabs after ongoing neurodegeneration could give more insight in the role of autoabs in glaucoma pathogenic mechanisms and might help to understand the differences in the enormously complex batch of given autoab reactivities.

## 2. Material and Methods

### 2.1 Patients

**Fig 1:**
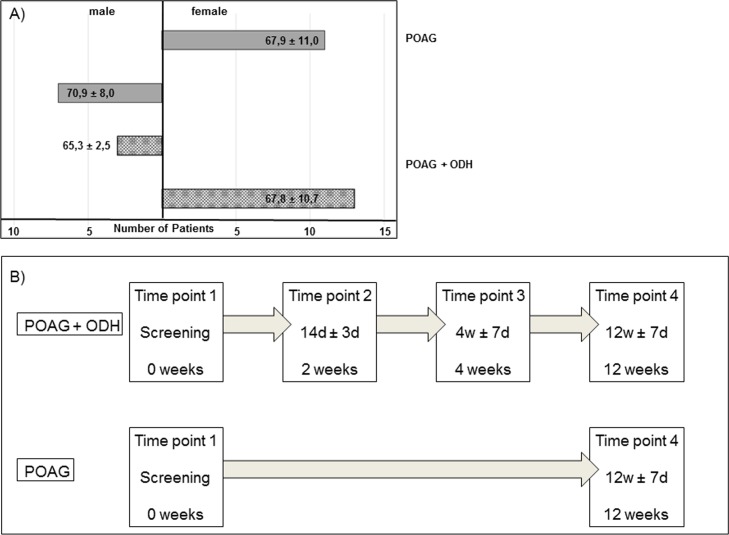
Age- and gender-distribution in patient cohort and sampling time points. A) The POAG + ODH group consists of 16 patients. Among these are 13 female persons and 3 male subjects with an overall mean age of 67.4 ± 9.7 years. For POAG group 11 female and 7 male patients were included. These 18 patients had an overall mean age at study inclusion of 67.4 ± 9.7 years. B) Blood collection and subsequent serum isolation was carried out at the beginning (Screening, 0 weeks) and at the end of the study (12 weeks) for both patient groups. POAG + ODH patients had additional sampling time points 14 days ± 3 days and 4weeks ± 7days after time point 1.

16 patients with POAG and ODH and 18 patients with POAG were included in the study and classified in accordance with the guidelines of the European Glaucoma Society (Details Fig 1A). All participants were subject of a full ophthalmologic examination and treatment at the Department of Ophthalmology, University Medical Center Mainz. Examination, measurement of visual acuity and blood collection time points were directly after study inclusion (0 weeks, time point 1: T_1_) and after 2 weeks ± 3 days (time point 2: T_2_), 4 weeks ± 7 days (time point 3: T_3_) and 12 weeks ± 7 days (time point 4: T_4_) for the POAG + ODH group and at time point 1 and time point 4 for the POAG group (Schematic overview Fig 1B). To consider naturally occurring variations of protein content in serum samples, blood tube collection took place in the same time range for each patient. Collection of samples and examination were performed corresponding the 1964 Declaration of Helsinki on biomedical research involving human subjects and approved by the ethics committee of the Landesärztekammer of Rhineland-Palatinate. Before onset of the study written informed consent was taken from all study participants.

### 2.2 Serum samples

Blood samples were collected at each time point using blood collection tubes (Sarstedt, Germany). After centrifugation (10 min, 10°C, 4000 rpm), serum was aliquotated and stored at -80°C for subsequent analysis. Immediately prior to microarray analysis samples were thawed up and diluted 1:250 in phosphate buffered saline (PBS). To analyze autoab in serum samples each sample was incubated once on a microarray slide. Each ab-level for a single antigen was therefore determined in parallel in individual samples. All serum samples included in the study were processed on the microarray slides on the same day according to the procedure described below.

### 2.3 Protein microarray

Antigens of interest (S2 Table) were spotted on nitrocellulose coated glass slides by the use of a non-contact array spotter (sciFLEXARRAYER S3, Scienion, Germany). In total 57 different, highly purified commercially purchased proteins were used and spotted as three technical replicates in line onto each of the 16 subarrays per slide. Spot position and volume were monitored via camera-assisted software (sciDrop-Volume, autodrop-detection). All incubation steps were carried out under slight agitation at 4°C and pre-spotted slides were covered with 16-pad FAST frame hybridization chambers (Whatman, Maidstone, UK). After blocking unspecific binding sites of nitrocellulose membrane (0.5% bovine serum albumin in 0.5% Tween 20 in PBS (PBS-T), 1 h) and three washing steps (10 min each time with 100 μl PBS-T), study samples were incubated randomly for 16 h. Slides were washed again (3 times, 10 min, 100 μl PBS-T) and incubated with a fluorescent labeled secondary ab (anti-human IgG H+L) for 1 h in the dark. Final washing steps (each 15 min) once with PBS-T and twice with ultrapure water were done. After vacuum-drying (Speed Vac, Thermo), emitted fluorescence signals were digitized by scanning the slides with a high-resolution confocal microarray scanner (Affymetrix 428^TM^ Array Scanner, Wycombe, UK). Incubation with PBS served as negative control. The used protein-microarray approach was established previously. The intra- and inter-slide variability for this platform was tested and described elsewhere [16, 27].

### 2.4 Data analysis

Spot intensity of each single spot was calculated by subtraction of local background intensity from median intensity value. Before statistical analysis of array data, raw intensities were normalized with a constant scale factor and technical replicates were averaged. Technically faulty spots were not considered for the final analysis. In detail, spots below size threshold, unexpectedly empty spot positions and spots separated into two individual spots were excluded from the final data set. Additionally, spots with a visible contamination were not considered, because a correct digitalization of intensity values of these spots with the used software cannot be ensured. In the present study an average percental proportion of 3% of spots were excluded from the analysis per incubated subarray (5 spots of 171 analyzed spots in total per serum sample). Linear regression analysis was performed for the POAG + ODH group with mean intensity values of each ab-level at each time point by using a linear regression model. R- values ≥ 0.8 were considered as relevant. Further statistical analysis were conducted using Statistica 12.0 (Statistica, Tulsa, AZ, USA). Intra- and inter-group comparisons of time point 0 weeks and time point 12 weeks were compared with Student’s t-test. No adjustments for multiple testing was done. Thus, mentioned significant p-levels should be considered as remarkable low p-levels. Detected alterations should guide to hypotheses generation and should be reanalyzed in further confirmative studies.

**Fig 2:**
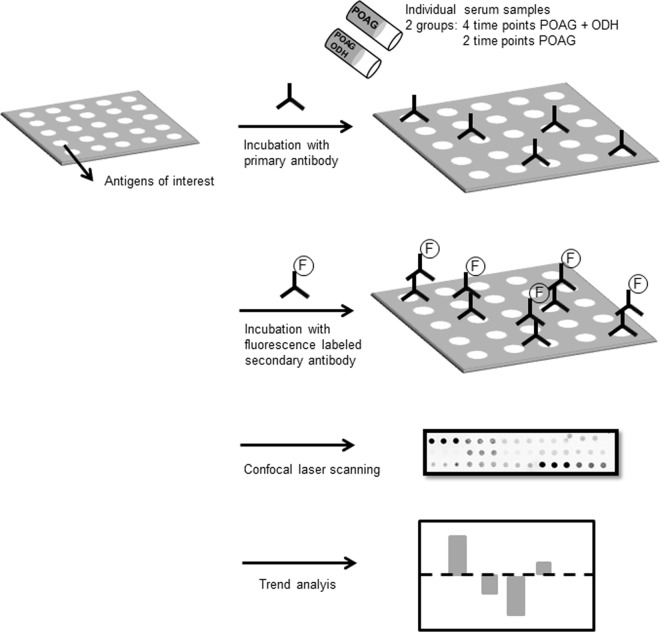
Schematic overview of microarray workflow. Shown is the basic procedure of ab-screening. Nitrocellulose-slides with antigens of interest are incubated with patients´sera in order to capture autoabs. For visualization of the resultant antigen-ab complexes, slides are incubated with fluorescence-labeled (F) secondary abs followed by confocal laser scanning. Measured intensities were normalized and semi-quantitative results were further investigated by linear trend and statistical analysis.

Graphical overview of complete workflow is shown in Fig 2.

## 3. Results

**Fig 3:**
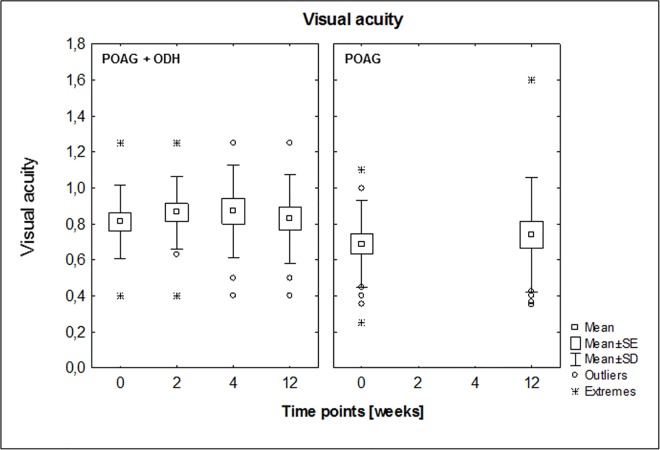
Visual acuity in sample cohort over study period. No significant changes could be shown, neither in intra- nor in inter-group comparisons.

**Fig 4:**
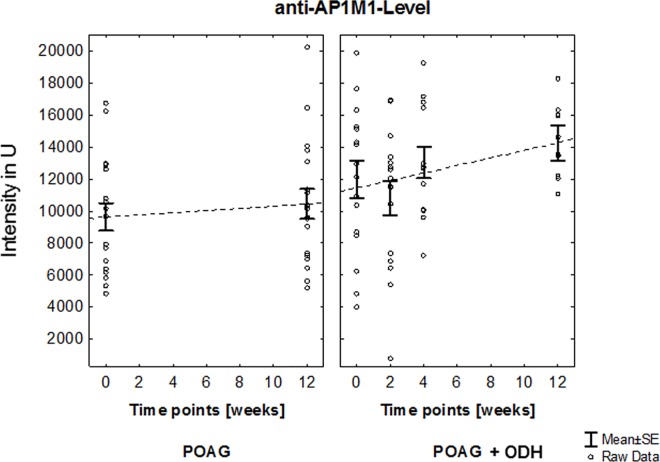
Longitudinal trend analysis of anti-AP1M1 level. Linear increment of ab-level after study inclusion in POAG + ODH patients with r-value ≥ 0.8 could be shown. Referring to time point 1 an increase of 19% in ab-level could be detected at the end point of the study at time point 4 (12 weeks after screening time point). In contrast, no relevant changes could be detected in the POAG group in the same time period. Additionally, time point comparison between study groups revealed statistically significant higher level of anti-AP1M1-level in POAG patients with ODH at time point 4 (p = 0.01,Student’s t-test).

**Fig 5:**
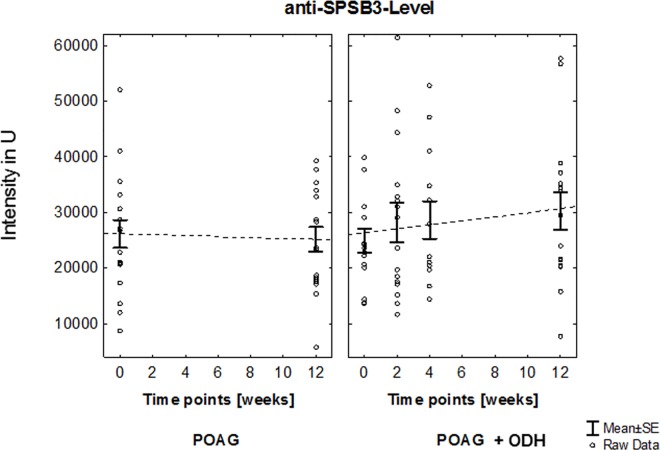
Longitudinal trend analysis of anti-SPSB3 level. Linear increment of ab-level after study inclusion in POAG + ODH patients with r-value ≥ 0.8 could be shown. Referring to time point 1 an increase of 22% in ab-level could be detected at the end point of the study at time point 4 (12 weeks after screening time point). In contrast, no relevant changes could be detected in the POAG group in the same time period.

This protein microarray analysis focused on autoab repertoire of POAG patients with ODH (affected eye: right: n = 12, left: n = 4). Visual acuity of each patient was determined at each sampling time point. No statistically significant changes could be detected, neither in inter- nor intra-group comparisons (POAG + ODH: affected eye values vs POAG: mean values, Fig 3). Autoab reactivities were analyzed by using protein microarray slides prespotted with 57 antigens representing different groups of proteins e.g. heat shock proteins, ocular and neuronal antigens or antigens involved in signal transduction. In previous studies part of these antigen panel was used to detect immunoreactivities in serum an aqueous humor of patients with POAG and healthy subjects [16]. Overall we were able to detect complex autoab pattern in all samples at each study time point in patients with POAG and ODH and also in POAG group. With respect to the immunoreactivities at time point 1 we were able to identify changing level of anti-adaptor protein 1 (AP-1) complex subunit mu-1 (AP1M1)-level (Q9BXS5 [28], Fig 4) and anti-SPRY domain-containing SOCS box protein 3 (Q6PJ21 [28], SPSB3)-level (Fig 5) over the study period in the POAG + ODH group with respect to the POAG group. Regarding longitudinal trend analysis with a linear regression model we were able to detect a linear increment of ab-level after study inclusion in POAG + ODH patients with r-values ≥ 0.8 (anti-AP1M1 ab, anti-SPRY ab: r = 0.84; R^2^ = 0.7). Referring to time point 1 an increase of 19% (anti-AP1M1) and 22% (anti- SPSB3) in ab-level could be detected at the end point of the study at time point 4 (12 weeks after screening time point). In contrast, no relevant changes could be detected in the POAG group in the same time period. Additionally, time point comparison between study groups revealed statistically significant higher level of anti-AP1M1-level in POAG patients with ODH at time point 4 (p = 0.01,Student’s t-test). Normalized signal intensity values for each patient (POAG and POAG + ODH) at each time point for anti-AP1M1 and anti-SPSB3 Ab are shown in S1 Table.

## 4. Discussion

The main target of therapeutic approaches in glaucoma is elevated IOP and the protective role of IOP-lowering is well known and documented in the large clinical glaucoma trials such as the Early Manifest Glaucoma Trial, the Collaborative Normal Tension Glaucoma study or the Advanced Glaucoma Intervention Study. Reduced visual field defect progression in clinically manifest glaucoma is a benefit of medicamentous reduction of IOP for many patients [23, 29–31]. Disease progression is therefore an important indicator for therapy advancement or adaption to achieve the best possible result for each patient. Thus, a major point in successful glaucoma disease management is the accurate identification of structural defect progression to restore the individual vision-related quality of life and develop an effective management plan in each glaucoma case. Accurate relationship between structural progression and functional deterioration is not yet elucidated and this makes detection of progression and risk assessment in clinical routine challenging. Measurements of structural defects with standard techniques do not always correlate with and are not predictive for individual functional outcome of patients especially in early disease stages. Also in the present analysis no changes in visual acuity over study period could be detected. However it is shown that ODH can be indicator for glaucoma progression and a significant risk factor to obtain diminished visual function [32–35]. Therefore the analyzed study population could be useful to find other markers that support clinically suspect cases such as serum autoantibodies as part of the immune response to the ongoing progression indicated by ODH in patient’s eyes. Autoantibodies as possible additional component of glaucoma pathogenesis are discussed in recent years. In several studies with autoimmune glaucoma animal models an increase of autoreactive abs due to antigen immunization which lead to RGC loss and ab deposits in retinal structures could be shown [36–38]. Also in patients different autoab-profiles between glaucoma groups and IgG staining in human glaucomatous retinae could be detected [12, 15, 16, 39]. But the function of the enormously complex autoab repertoire is still an unanswered question. An interesting point to elucidate is if autoabs has to be considered as part of the pathogenesis of the disease or as a sequelea to glaucomatous changes. To our knowledge this study reports the first analysis of autoab reactivities in POAG patients with recently diagnosed ODH. In the analyzed time period of 12 weeks we were able to detect two autoabs with increased level in serum of patients with ODH in comparison to levels in patients with chronic POAG without ODH as a sign for glaucomatous progression. Target protein of anti-AP1M1 ab is one of four subunits (mu 1, (μ1)) of clathrin adaptor protein complex 1 (AP1). Together with six other known AP complexes it belongs to a family of heterotetrameric complexes in mammals which are regulators of vesicular transport and protein sorting [40, 41]. Ubiquitously expressed AP1 with subunit isoform μ1A is involved in transmembrane trafficking and is found on the trans-Golgi-network (TGN) and endosomes. AP1 containing isoform μ1B are expressed in some subgroups of polarized epithelial cells [42]. Additionally an association with clathrin coated vesicles, which are involved in intracellular transport and secretory processes of cargo proteins, is shown. APs recruit clathrin and serve as anchors for polymerization of this molecule. AP1s bind to cytoplasmatic parts of cargo proteins and thereby connect them to the clathrin matrix [43, 44]. Thus, also the mechanism of protein sorting, e.g. of transmembrane proteins or receptors, from one location in the cell to another is mediated via APs. For example sorting of transmembrane receptors in neurons or transport of amyloid precursor protein, which plays a major role in Alzheimer’s disease, is supported by AP1s [45–47]. Another ab, with elevating level in patients with POAG + ODH is anti- SPSB3. Besides SPSB1, SPSB2 and SPSB4, SPSB3 belongs to the family of SPRY-containing suppressors of cytokine signaling (SOCS) box proteins. The SPRY (SpIa/Ryanodine receptor)-domain is a common protein domain in eukaryotes which is involved in various protein-protein interactions by functioning as adaptor or scaffold for example in immune-system-related processes [48]. The SOCS box is a structural domain of 40-amino acids and is found in a superfamily of more than 70 human proteins of nine different families. One of the main functions of this C-terminal SOCS box is the recruitment of E3 ubiquitin ligase complexes by serving as a substrate recognition module. Together with E1 ubiquitin-activating enzyme and E2 ubiquitin-conjugating enzyme, E3 ligase complex mediates one of the major post-translational modifications, so called protein ubiquitination. This process is essential for numerous intracellular pathways for example internalization and trafficking of receptor proteins, antigen presentation, apoptosis and proteasomal degradation [49–51]. Dysregulation in genes of E3 ubiquitin ligase family leads to disturbance in generation of autoab response by impact on clonal anergy or differentiation of regulatory T-cells. And thus can have implication in development of autoimmunity [52]. Also for glaucoma, precisely POAG a candidate gene of the SOCS box protein family is known. Recent studies showed possible role for Ankyrin repeat and suppressor of cytokine signaling box containing protein-10 in ubiquitination processes in trabecular meshwork cells [53]. Additionally, SOCS box proteins play a role in cytokine signaling via negative regulation of signal transduction from the Janus kinase (JAK)-associated cytokine receptor complexes (CRCs). By marking of activated CRCs for ubiquitin mediated proteolysis SOCS box proteins are able to regulate innate and adaptive immune response [49–51]. Glaucoma is also linked to JAK-Signaling and studies showed that that participating pathways mediate RGC survival in rat eyes after IOP-elevation [54, 55]. Even if the detailed function or mechanistic influence of autoabs targeting AP1M1 or SPSB3 is unknown, autoabs might influence physiological protein function or subsequent signaling cascades by activating or inhibiting properties [56]. Increasing level might reflect ongoing subclinical structural changes or cellular processes and may be a hint for an immune response to apoptotic RGC loss. The results indicate that natural balance of autoab repertoire may be disturbed through ongoing glaucomatous progression during ODH.

By the use of advanced antigen-microarray platform this study provides a first longitudinal insight into serum immunoreactivity in POAG patients with ODH. To our knowledge, this is the first pilot study to address the question whether or not autoabs are relevant to glaucoma progression. Hints are found for changes in autoab repertoire of patients with ODH as sign of ongoing deterioration of glaucoma disease processes. The variability of autoab-repertoire among individuals is well known and makes detection of alterations over time challenging, especially in a small sample cohort. Patients with POAG and ODH are rare and the recruitment in clinical routine is challenging. Also the introduction of a positive control, like for example GAPDH expression level for estimation of protein-level in western-blot analysis is quite difficult, but should be considered for further analysis. Due to enormous complexity of and various influence on autoab-repertoire a suitable control for this explorative study was not realizable. Quality control (QC) in the field of ab-profiling by means of microarray is a growing scope, but a standard procedure for data evaluation is not available yet and QC implementation in the routine of protein-microarray processing is still considered as underdeveloped [57, 58]. Another challenge of explorative data analysis in bulk, is the statistical correction for multiple testing in the style of e.g. Bonferroni. This adjustment corrects the increased risk of a type I error when multiple comparisons in parallel are conducted [59, 60]. The usability and necessity is discussed controversial for example by Perneger *et al*. and the routine use of Bonferroni correction is considered as dependent on the intention of the investigator in each experimental setup [60, 61]. Therefore we passed on the mentioned adjustments and consider the detected alterations in anti-AP1M1 ab and anti-SPSB3 ab over time in POAG + ODH as valuable hints to answer the question whether or not autoabs are relevant to glaucoma progression. The focus of this pilot study was to discover potential differences in autoab-formation between POAG patients with and without ODH. To consider them as a sign of proceeding glaucomatous changes, corresponding ab should not alter in the group of POAG patients without ODH. Only the two described autoab-reactivites are in line with the focused hypothesis. Analyzed by the described linear regression, other autoab-level show no relevant differences between POAG and POAG + ODH over time. Following the classic approach for detection of markers after the discovery phase, here realized with an immunoproteomic platform, subsequent confirmative tests to validate ab-reactivities should be done [62]. Results of this exploratory study should be reanalyzed with more serum samples and abs of interest should be screened for example in retrospective and prospective studies to evaluate their value in glaucoma pathogenesis [63].

Despite the described limitations of the study, the observed trends might give further insight in the role of autoabs in GON and might represent useful markers for future screening tools and therapeutic intervention strategies. The detected alterations in this pilot study should be validated in a larger patient cohort in the future.

## Supporting Information

S1 Table Normalized intensity values for each patient (POAG and POAG + ODH) at each time point for anti-AP1M1 Ab and anti-SPSB3 Ab. (XLSX)

S2 Table List of analyzed proteins. (XLSX)
